# Negative pressure wound therapy for preventing wound complications following stoma reversal: A systematic review and meta-analysis of randomized controlled trials

**DOI:** 10.1016/j.apjon.2025.100778

**Published:** 2025-08-26

**Authors:** Siqing Li, Xiaofang Guan, Xirong Huang, Zhong Wang, Linjie Mo, Minyi Xie, Yuxia Liu, Wenxin Luo

**Affiliations:** a Department of Gastrointestinal Surgery, The Fifth Affiliated Hospital, Sun Yat-sen University, Zhuhai, China; b Nursing Department, Shenzhen Futian District Maternity & Child Healthcare Hospital, Shenzhen, China; c Department of General Surgery, The Fifth Affiliated Hospital, Sun Yat-sen University, Zhuhai, China

**Keywords:** Wound complications, Negative pressure wound therapy, Stoma reversal, Meta-analysis, Surgical site infections

## Abstract

**Objective:**

To evaluate the effectiveness of negative pressure wound therapy in preventing wound complications following colorectal stoma reversal and provide evidence-based recommendations for its clinical application.

**Methods:**

A comprehensive search was conducted across five English databases (PubMed, Embase, Web of Science, Cochrane Library, Scopus) and three Chinese databases (Chinese Biomedical Literature Database, China National Knowledge Infrastructure, Wanfang) from their inception to December 2024. Grey literature and trial registries were also included. Eligible studies were randomized controlled trials investigating negative pressure wound therapy for patients undergoing stoma reversal. Primary outcomes included wound complications (surgical site infections, hematoma, seroma); secondary outcomes comprised hospital stay, wound healing duration, and dressing costs.

**Results:**

Eight randomized trials involving 517 patients were included. Negative pressure wound therapy significantly reduced wound complications (odds ratio, 0.50; 95% confidence interval, 0.27–0.94), shortened wound healing time (mean difference, −4.31; 95% confidence interval, −4.97 to −3.65), and decreased hospital stay (mean difference, −1.05; 95% confidence interval, −1.70 to −0.41). Subgroup analysis demonstrated benefits in the linear closure group (odds ratio, 0.11; 95% confidence interval, 0.02–0.49) but not in the purse-string closure groups. No significant differences were observed in surgical site infections, hematoma, seroma, or dressing costs.

**Conclusions:**

Negative pressure wound therapy reduces wound complications, accelerates wound healing, and shortens hospital stays after stoma reversal. Standardized protocols and additional high-quality trials are required to establish negative pressure wound therapy as a routine practice.

**Systematic review registration:**

Prospectively registered in PROSPERO (ID CRD42023451001).

## Introduction

A temporary stoma created during colorectal surgery to mitigate postoperative complications is typically closed within 3–6 months of the initial procedure. While stoma reversal surgery offers the potential to restore bowel continuity, it carries a substantial risk of abdominal surgical complications, including wound complications. For clarity, wound complications are defined here as adverse events affecting the surgical incision within 30 days postoperatively, encompassing surgical site infections (SSI), hematoma, seroma, wound dehiscence, and delayed healing. Other common complications include intestinal obstruction, incisional hernia, and anastomotic leakage.^1^ SSI is defined in accordance with the Centers for Disease Control and Prevention (CDC) criteria: an infection involving the surgical incision (superficial, deep, or organ/space) confirmed by clinical signs (e.g., redness, warmth, pus) or laboratory testing, occurring within 30 days of surgery (or within 1 year if a prosthetic implant is present).^2^ SSI is the most prevalent and clinically significant complication of stoma reversal, with incidence rates ranging from 2% to 41% across studies.^3^, ^4^, ^5^ This elevated risk may stem from microbial colonization at the stoma site and intraoperative contamination during bowel manipulation, classifying the closure site as a clean-contaminated wound.^6^

SSI-associated morbidity extends beyond infection management, potentially leading to incisional herniation, delayed wound healing, prolonged hospitalization, increased healthcare costs, and diminished patient satisfaction and quality of life.^7^^,^^8^ Despite advancements in infection control strategies, such as antimicrobial-coated sutures, silver-based dressings, plasma-mediated surgical tools, purse-string closure techniques, and iodine-impregnated drapes, the cumulative SSI risk in stoma reversal procedures remains clinically significant.

Negative-pressure wound therapy (NPWT), a well-established modality for postoperative wound management, has gained widespread clinical acceptance across diverse wound types due to its putative infection-prevention properties.^9^, ^10^, ^11^ This therapeutic approach employs a sealed dressing system connected to a vacuum device that applies controlled subatmospheric pressure, facilitating exudate removal while potentially enhancing angiogenesis, reducing tissue edema, and improving wound tensile strength - mechanisms hypothesized to accelerate healing and mitigate infection risks.^12^^,^^13^ NPWT has been extensively studied in various surgical contexts, including orthopedic, cardiothoracic, and abdominal surgeries, with mixed results regarding its efficacy in reducing SSIs.^9^^,^^10^ In colorectal surgery, NPWT has shown promise in reducing wound complications, particularly in high-risk patients, but its application in stoma reversal remains controversial due to inconsistent findings and limited high-quality evidence.^14^^,^^15^

Despite its prevalent clinical adoption, significant outcome discrepancies persist across studies. Particular controversy surrounds NPWT's efficacy in reducing SSIs following stoma reversal. Notably, some studies have reported comparable SSI rates between NPWT and conventional dressings in these cases. However, NPWT demonstrated secondary benefits—including enhanced wound healing trajectories, reduced postoperative pain, and superior cosmetic outcomes.^16^^,^^17^ These findings underscore the need for large-scale randomized controlled trials (RCTs) to establish definitive evidence regarding NPWT's therapeutic value in this surgical context.

Current evidence synthesis remains limited by methodological constraints. Although AlJoaib et al.^14^ systematically evaluated NPWT in colorectal surgical closures, their analysis incorporated only four RCTs specific to stoma reversal. Similarly, Zhu et al.^15^ conducted a recent review spanning 2016–2022 publications but included non-randomized studies, potentially compromising the evidence hierarchy. This scarcity of comprehensive quantitative synthesis, coupled with inconsistent guideline recommendations, has created barriers to clinical implementation and theoretical ambiguity regarding NPWT's prophylactic efficacy in primarily closed incisions.

To address these evidence gaps, our systematic review and meta-analysis pursued three primary objectives: (1) to critically appraise and synthesize existing clinical evidence, (2) to quantitatively assess NPWT's effectiveness in preventing SSIs and other wound complications following colorectal stoma reversal, and (3) to establish an evidence-based framework for guiding future clinical practice and research initiatives. By incorporating a larger number of RCTs and focusing specifically on stoma reversal, this study aims to provide a more robust effect estimate and clarify the role of NPWT in this surgical context.

## Methods

This systematic review adhered to the Cochrane Handbook for Systematic Reviews of Interventions for methodological guidance.^18^ Reporting followed the PRISMA 2020 statement.^19^ The protocol was registered in the International Prospective Register of Systematic Reviews (PROSPERO registration number: CRD42023451001).

### Data sources and search strategies

A comprehensive literature search was conducted using five English-language databases (PubMed, Embase, Web of Science, Cochrane Central Register of Controlled Trials, and Scopus) and three Chinese-language databases (Chinese Biomedical Literature Database, China National Knowledge Infrastructure, and Wanfang Database) from their inception to December 2024. Grey literature was manually searched via the European Association for Grey Literature Exploitation (http://www.opengrey.eu), clinical trial registers (https://clinicaltrials.gov), and the WHO International Clinical Trials Registry Platform search portal (https://www.who.int/ictrp/search/en/). Filters were applied based on study type, publication date, and research-related keywords.Additionally, references of included articles and reviews were manually screened to identify relevant grey literature missed in initial database searches.

To ensure appropriate study selection based on design, we used the SIGN filters (https://www.sign.ac.uk/using-our-guidelines/methodology/search-filters/). For RCTs, we applied SIGN filter criteria specific to RCT retrieval, including search terms and limits designed to accurately capture RCTs from databases-enhancing the validity of our meta-analysis. The search strategy utilized Medical Subject Headings (MeSH) and free-text terms, including: (“Negative-Pressure Wound Therapy” OR “Negative Pressure Wound Therapy” OR “Vacuum-Assisted Closure”) AND (“ileostomy closure” OR “ileostomy reversal” OR “stoma reversal” OR “stoma closure”). The complete search strategy is provided in Supplementary Table S1-8.

### Eligibility criteria

Eligible articles were selected using the Participants, Interventions, Comparisons, Outcomes, and Study Design (PICOS) model.^18^ Inclusion criteria were: (1) Population: Adults (≥ 18 years) with colorectal cancer who underwent stoma reversal. (2) Intervention: NPWT, defined as use of a vacuum-assisted device for wound treatment following stoma reversal surgery. (3) Comparison: Studies comparing NPWT with conventional wound treatments (e.g., iodoform or sterile gauze dressing) or no intervention. (4) Outcomes: Incidence of wound complications (including SSIs, hematoma, and seroma), measured using standardized scales; secondary outcomes included length of hospital stay, wound healing duration, and total cost of wound dressings (all measured using standardized scales). (5) Study Design: RCTs, including crossover, cluster, and pilot studies.

Studies were excluded if patients had: (1) Allergies interfering with NPWT application or evaluation. (2) Comorbidities strongly suspected to affect wound healing (e.g., familial adenomatous polyposis, inflammatory bowel disease, hemodialysis, poorly controlled diabetes mellitus [glycated hemoglobin ​≥ ​7.5%], or steroid use). (3) Difficulty in dressing changes due to dementia, psychiatric illness, or postoperative delirium. Excluding these patients may introduce selection bias (as such conditions are common in older adults and could influence wound healing), but this was necessary to ensure feasible NPWT application and accurate outcome assessment.

Additionally, protocols, reviews, conference abstracts, case reports, non-randomized trials, duplicates, ongoing studies without results, and studies lacking outcome measures were excluded. For studies with incompletely reported data, corresponding authors were contacted via email to request missing information.

### Study selection and data extraction

The study selection process was conducted systematically using EndNote X9 (Clarivate Analytics, Boston, MA, USA) for reference management and duplicate removal. Two independent investigators screened records by title and abstract against predefined eligibility criteria, followed by full-text assessment of potentially eligible studies. Data extraction was performed independently by the same reviewers using standardized forms, capturing: (1) study identification (author, region, publication year, setting); (2) participant characteristics (demographics, sample size, stoma classification, wound closure technique); (3) intervention details (NPWT specifications: device type, pressure settings, treatment duration, frequency); and (4) outcome measures (assessment methods for primary and secondary endpoints).

Inter-rater discrepancies at any stage were resolved via consensus with a third investigator. The study selection workflow, including exclusion reasons, was documented per PRISMA guidelines^19^ and presented in the corresponding flow diagram.

### Risk of bias assessment

Using the Cochrane Risk of Bias 2 (RoB 2) tool,^20^ which evaluates five domains (bias from randomization, deviations from intended interventions, missing outcome data, outcome measurement bias, and selection bias in reported results) as “low risk”, “some concerns”, or “high risk”, two reviewers independently assessed risk of bias. Disagreements were resolved with a third reviewer.

### Data synthesis and analysis

Both quantitative meta-analysis and descriptive synthesis were used. Qualitative summaries and detailed tabulations of study characteristics were conducted. Statistical analyses were performed using R software (version 4.0.3; R Foundation for Statistical Computing, Vienna, Austria). For categorical outcomes (e.g., wound complications, SSIs, hematoma, seroma), odds ratios (ORs) and 95% confidence intervals (CIs) were calcuated. For continuous outcomes, mean differences (MDs) or standardized mean differences (SMDs) with 95% CIs were used. Wound healing time was defined as days from stoma reversal to complete epithelialization of the surgical incision, with no residual inflammation (e.g., redness, swelling), drainage, or infection. Other continuous outcomes included hospital stay and total cost.

Heterogeneity was evaluated using *I*^*2*^ statistics. If *I*^*2*^ > 50% or ≥ 2 PICO-SD domains showed material variations (e.g., mixed VAC/PICO devices with stoma closure techniques), a random-effects model was used.^18^ Subgroup and sensitivity analyses explored potential sources of significant heterogeneity (*I*^*2*^ ​> ​50%).

Subgroup analyses were conducted by stoma closure techniques (purse-string closure [PSC] vs. linear closure [LC]) and NPWT device type (e.g., VAC, PICO) to identify effect modifiers. Sensitivity analyses evaluated result robustness by excluding studies with imputed statistics or high risk of bias. When > 20% of studies lacked essential statistics (e.g., standard deviations [SDs]), multiple imputation was used to mitigate selection bias: predictive mean matching (PMM) for continuous outcomes (using observed group means/SDs from complete studies); multinomial logistic regression for dichotomous outcomes (based on treatment-arm event probabilities).

## Results

### Search results

**Fig. 1:**
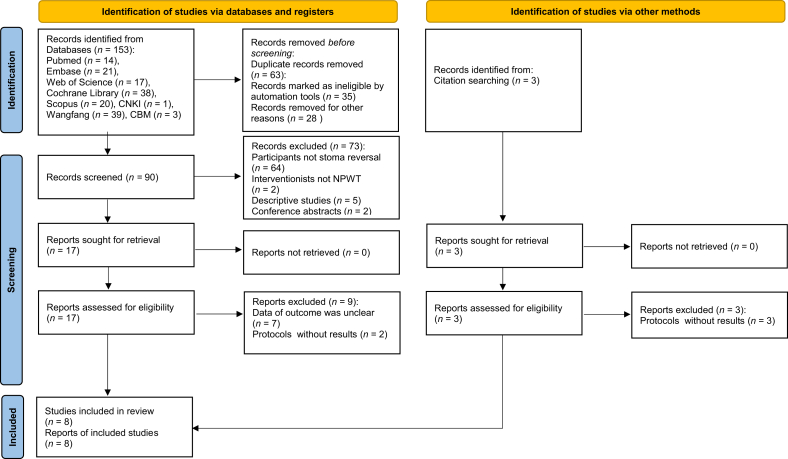
Flow diagram illustrating the original process of screening and identification of studies.

The systematic search identified 156 potential records: 153 from electronic databases and 3 from gray literature. After removing 63 duplicates, 93 records underwent title and abstract screening; 73 were excluded for failing to meet inclusion criteria. Full-text review of the remaining 20 articles yielded 8 eligible RCTs for final inclusion (Fig. 1).

### Studies characteristics

The eight included RCTs, published between 2016 and 2024, represented diverse regions: two from China,^21^^,^^22^ two from Japan,^17^^,^^23^ and one each from Italy,^16^ Poland,^24^ South Korea,^25^ and Australia.^26^ Collectively, they enrolled 517 patients with stoma reversal, with balanced randomization (NPWT: *n* ​= ​259; control: *n* ​= ​258).

Wound closure techniques varied: six employed PSC,^16^^,^^17^^,^^23^, ^24^, ^25^, ^26^ and two implemented LC.^21^^,^^22^ Intervention durations ranged from 3 to 14 days, with follow-up periods of 16–42 days. Detailed characteristics are presented in Table 1.

**Table 1:** Characteristics of included studies.

Study (author, year)	Country	Sample E/C	Age (years) E/C	Gender (M:F) E/C	BMI (kg/m^2^) E/C	Stoma type (Ileostomy: Colostomy) E/C	Wound closure	Intervention device/pressure/duration	Control	Follow-up time (days)	Observation indicators
Carrano et al., 2021	Italy	50/48	56.32 ​± ​12.92/55.08 ​± ​16.25	35:15/31:17	23.81 ​± ​3.38/23.45 ​± ​3.66	41:9/38:10	PSC	NPWT (PICO-7TM) /80 ​mmHg ​± ​20 ​mmHg /7 (4–7) days	Iodoform gauze dressing	30	a, b, c, d, g
Wierdak et al., 2021	Poland	35/36	61.6 ​± ​11.3/62.4 ​± ​11.3	24:11/20:16	26.2 ​± ​4.5/26.2 ​± ​4.3	35:0/36:0	PSC	NPWT (NM) /NM /3 days	Gauze dressing	30	a, b, c, d, g
Kojima et al., 2021	Japan	Group B /Group C /Control 10/10/10	69 (48–75)/70 (43–84)/64 (48–82)	8:2/4:6/6:4	21.8 (16.5–23.8) /20.3 (17.0–26.2) /22.2 (16.4–30.1)	10:0 /10:0 /10:0	PSC	NPWT (PICO) /80 ​mmHg /7 or 14 days	Gauze dressing	16	a, b
Kang and Kim, 2023	South Korea	18/16	72 (32–87)/66.5 (33–78)	9:9/8:8	23.7 (15.2–31.2) /25.4 (18.7–31.9)	10:8/12:4	PSC	NPWT (PICO) /NM /7 days	Transparent waterproof dressing	30	a, b, e, f, g
Tiang et al., 2024	Australia	20/20	55.67 ​± ​17.1/62 ​± ​15.1	10:10/10:10	27.62 ​± ​6.21/26.12 ​± ​4.5	20:0/20:0	PSC	NPWT /125 ​mmHg /7 days	Absorbent cotton dressing	42	a, b
Uchino et al., 2016	Japan	28/31	48.1 ​± ​14.9/40.4 ​± ​15.9	17:11/23:8	19.8 ​± ​4.3/19.7 ​± ​3.8	28:0/31:0	PSC	NPWT (PICO) /NM /14 days	Adhesive plaster dressing	28	a, b, f
Xu et al., 2024	China	50/51	61.66 ​± ​14.22/64.43 ​± ​9.06	30:20/34:17	22.61 ​± ​3.06/21.82 ​± ​2.90	50:0/51:0	LC	MPNPWT /NM /3 days	Traditional dressing material	30	a, b, e, g
Cao et al., 2020	China	50/50	60.20 ​± ​14.70/62.80 ​± ​12.70	32:18/30:20	24.10 ​± ​3.80/22.90 ​± ​3.40	22:28/25:25	LC	SDCI-NPSD /99.76–149.64 ​mmHg /3 days	Gauze dressing	NM	a, b, f, g

E, experimental group; C, control group; Gender (M, male; F, female); BMI, body mass index; NM, not mentioned; PSC, purse-string closure; LC, linear closure; NPWT, negative-pressure wound therapy; MPNPWT, micro power negative-pressure wound therapy; PICO, Smith & Nephew Healthcare; SDCI-NPSD, subcutaneous drainage continuous irrigation negative pressure suction device. Continuous variables are presented as mean ​± ​standard deviation or median (range). Kojima: Group B (NPWT weekly use), Group C (NPWT fortnightly use). a: wound complications rates, including hematoma rate, seroma rate, and SSIs (surgical site infections) rate; b: SSI rate; c: hematoma rate; d: seroma rate; e: total cost for wound dressing; f: complete wound healing time; g: length of hospitalization.

### Risk of bias assessment

**Fig. 2:**
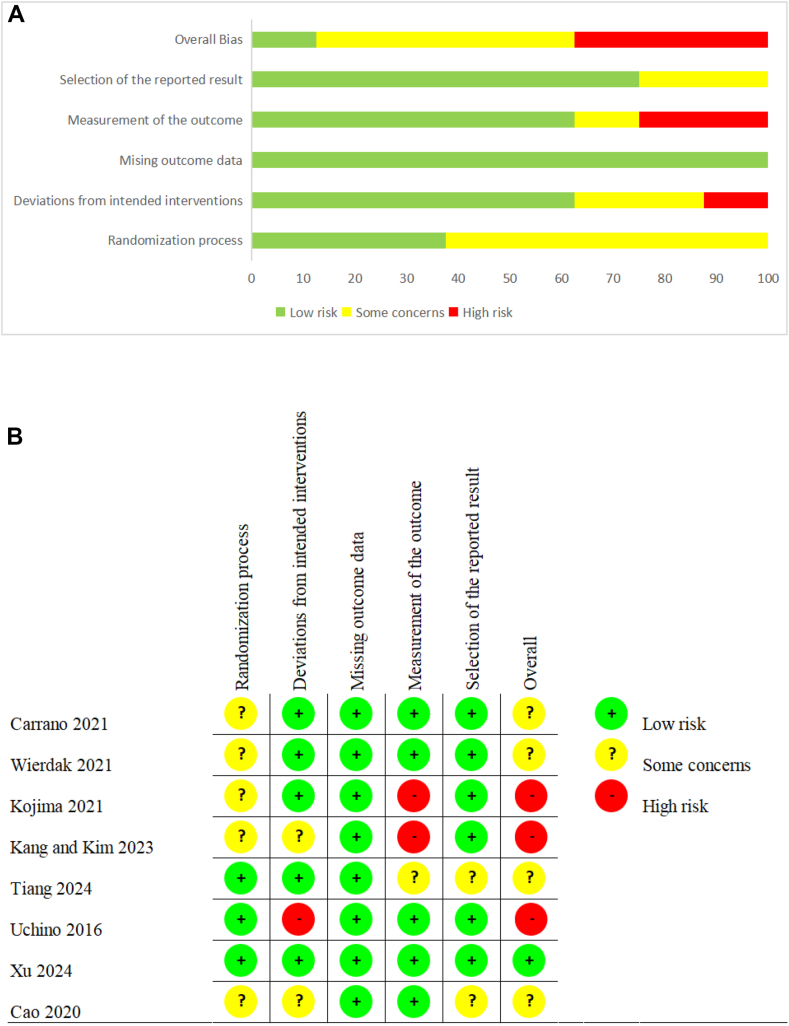
A, Risk of bias summary: the opinions of the review authors regarding each risk of bias item for every trial that was included. B, Risk of bias graph: review authors' judgements about each risk of bias item presented as percentages across all included studies.

Methodological quality of the included studies is graphically presented in Fig. 2. Overall, risk of bias was low to moderate, with some concerns regarding blinding of participants and personnel due to the nature of the intervention.

### Meta-analysis rResults

#### Wound complications

**Fig. 3:**
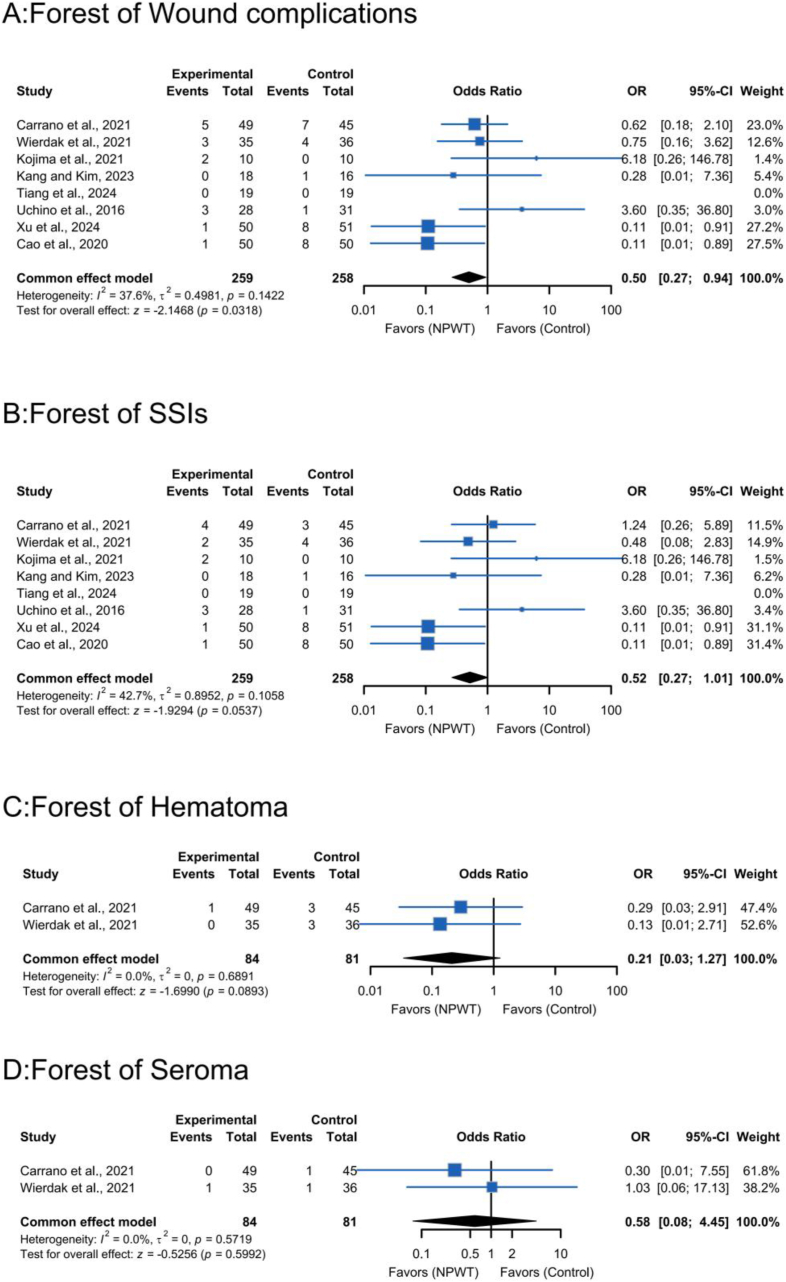
Meta-analysis on A, Wound complications; B, Surgical Site Infections (SSIs); C, Hematoma; D, Seroma.

Pooled analysis of eight RCTs^16^^,^^17^^,^^21^, ^22^, ^23^, ^24^, ^25^, ^26^ demonstrated superior efficacy of NPWT in reducing postoperative wound complications compared to standard dressings. Fixed-effects meta-analysis showed a statistically significant risk reduction (*OR*, 0.50; 95% *CI*, 0.27–0.94; *P* ​= ​0.03; Fig. 3A). Heterogeneity was low (*I*^*2*^ ​= ​37.6%; *τ*^*2*^_*3*_ ​= ​0.4981; *P* ​= ​0.14), indicating acceptable consistency across studies.

**Fig. 4:**
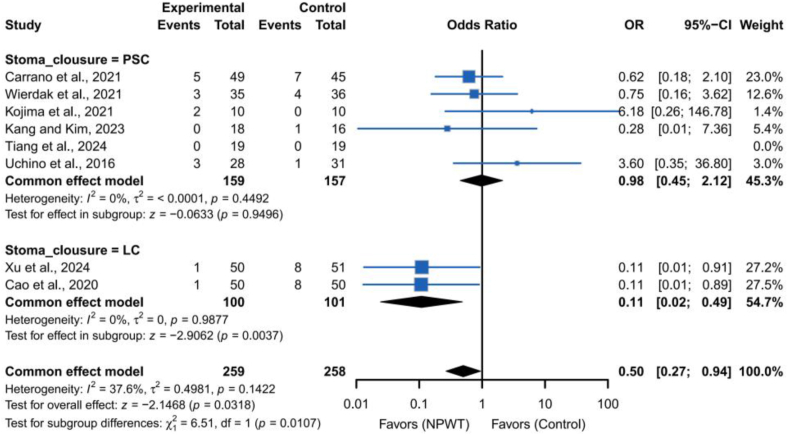
Forest plots depicting subgroup analyses categorized by various types of stoma closure techniques.

Notably, included studies varied in NPWT protocols: some used −125 ​mmHg, others −80 ​mmHg; treatment durations ranged from 3 to 14 days. These differences may contribute to observed heterogeneity, though it remained low. Subgroup analyses by wound closure technique revealed significant benefits in the LC group (*OR*, 0.11; 95% *CI*, 0.02–0.49), but not in the PSC group (*OR*, 0.98; 95% *CI*, 0.45–2.12) (Fig. 4).

#### SSIs

Pooled analysis of eight RCTs^16^^,^^17^^,^^21^, ^22^, ^23^, ^24^, ^25^, ^26^ (517 patients) showed no statistically significant reduction in SSIs incidence with NPWT compared to standard dressings (*OR*, 0.52; 95% *CI*, 0.27–1.01; *P* ​= ​0.05; Fig. 3B). Moderate heterogeneity was observed (*I*^*2*^ ​= ​42.7%; *τ*^*2*^_*3*_ ​= ​0.8952; *P* ​= ​0.11), within accepttable limits for fixed-effects modeling.

Comparator groups varied in standard dressings types (e.g., simple gauze vs. hydrocolloid) and dressing change frequencies, which might influence the outcomes, though their overall impact was limited.

#### Hematoma

Two studies^16^^,^^24^ reported hematoma outcomes, showing a non-significant trend toward reduced incidence with NPWT (*OR*, 0.21; 95% *CI*, 0.03–1.27; *P* ​= ​0.09; Fig. 3C). No homogeneity wass observed (*I*^*2*^= 0.0%; *τ*^*2*^_*3*_ ​= ​0; *P* ​= ​0.69). Notably, the two studies had different patient inclusion criteria (one focused on patients with specific pre-existing conditions, the other on a broader population).

#### Seroma

Analysis of seroma outcomes from two trials^16^^,^^24^ revealed no statistically significant difference between NPWT and standard dressings (*OR*, 0.58; 95% *CI*, 0.08–4.45; *P* ​= ​0.60; Fig. 3D). No homogeneity was observed (*I*^*2*^ ​= ​0.0%; *τ*^*2*^_*3*_ ​= ​0; *P* ​= ​0 .57). Standard dressings in these studies differed in composition, which might affect seroma formation, though no significant differences were found.

#### Economic evaluation

**Fig. 5:**
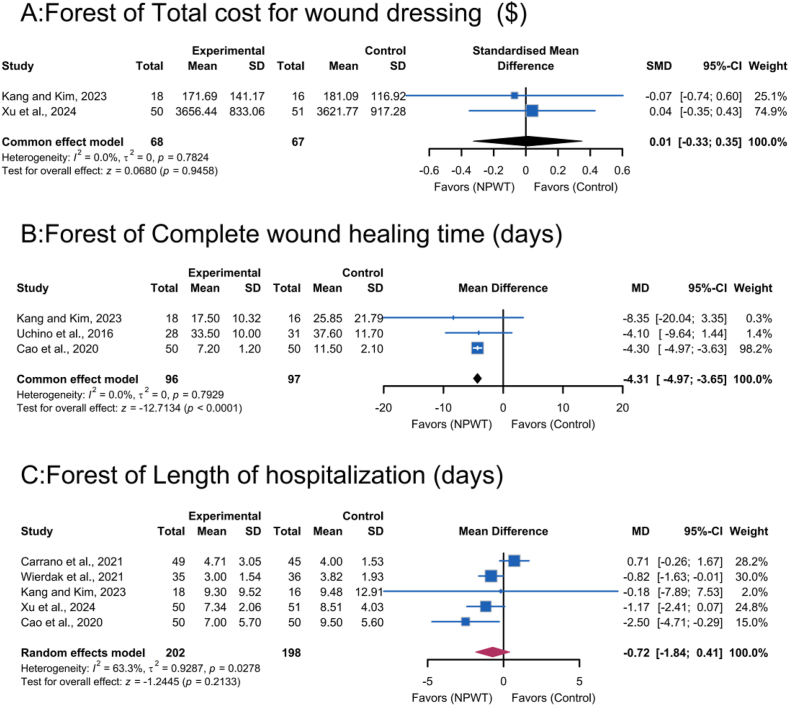
Meta-analysis on A, Total cost for wound dressing; B, Complete wound healing time; C, Length of hospitalization.

Two trials^21^^,^^25^ evaluated NPWT's economic impact, showing no significant difference in total wound dressing costs compared to standard dressing (*SMD*, 0.01; 95% *CI*, −0.33 to 0.35; *P* ​= ​0.95; Fig. 5A). No homogeneity was observed (*I*^*2*^ ​= ​0.0%; *τ*^*2*^_*3*_ ​= ​0; *P* ​= ​0.78). Economic evaluations were based on different healthcare cost structures, which might affect absolute cost values but not comparative results.

#### Wound healing time

Meta-analysis of three RCTs^17^^,^^22^^,^^25^ demonstrated that NPWT significantly accelerated wound closure compared to standard dressings, with a mean reduction of 4.31 days (95% *CI*, −4.97 to −3.65; *P* ​< ​0.0001; Fig. 5B). No homogeneity was observed (*I*^*2*^ ​= ​0.0%; *τ*^*2*^_*3*_ ​= ​0; *P* ​= ​0.79). NPWT devices varied in design/functionality but did not introduce heterogeneity in the wound healing time results.

#### Length of hospitalization

Pooled analysis of five RCTs^16^^,^^21^^,^^22^^,^^24^^,^^25^ initially showed no statistically significant reduction in hospitalization duration with NPWT compared to standard dressings (*MD*, −0.72; 95% *CI*, −1.84 to 0.41; *P* ​= ​0.21; Fig. 5C). Significant heterogeneity was observed (*I*^*2*^ ​= ​63.3%; *τ*^*2*^_*3*_ ​= ​0.9287; *P* ​= ​0.03), necessitating a random-effects model.

Excluding the study by Carrano et al.^16^ significantly reduced heterogeneity (*I*^*2*^ from 63.3% to 0.0%), with a more precise *MD* (−1.05; 95% *CI*, −1.70 to −0.41; *P* ​< ​0.0001), indicating a stable combined effect.

Observed heterogeneity may stem from variations in NPWT protocols (pressure settings: −80 ​mmHg vs. −125 ​mmHg; treatment durations: 3–14 days), patient populations, surgical techniques and postoperative care strategies in comparator groups.

## Discussion

### Main findings

This meta-analysis of RCTs evaluated NPWT's efficacy in reducing wound complications after stoma reversal. Key findings showed that NPWT significantly reduced wound complications, accelerated wound healing, and shortened hospital stays. Subgroup analyse indicated that NPWT's benefit in reducing wound complications was specific to the LC group.

These results align with prior syntheses^12^^,^^14^^,^^15^^,^^27^ and recent high-quality evidence supporting NPWT's efficacy in reducing wound complications with moderate certainty.^28^ This is corroborated by procedure-specific meta-analyses (e.g., colorectal surgery,^14^ abdominal wall closure^11^), reinforcing its potential as an evidence-based intervention in surgical wound care.

However, our results contrast with studies reporting no significant difference in wound complications rates between NPWT and standard dressings in gastrointestinal surgery.^28^^,^^29^ Discrepancies may stem from variations in study design (e.g., NPWT protocols: pressure settings, treatment durations), patient characteristics (e.g., comorbidities, surgical techniques), or broader study focuses.^11^^,^^30^ By focusing exclusively on stoma reversal and incorporating subgroup analyses, our study clarifies NPWT's specific value particularly for nursing practice by identifying a target population (LC patients) where NPWT may yield the greatest clinical benefit.

NPWT's differential efficacy across closure techniques may reflect its mechanobiological effects, which are particularly advantageous in larger, tension-prone wounds typical of linear closure.^31^^,^^32^ Linear closure involves a larger wound area and greater tissue tension than purse-string techniques. NPWT exerts cyclic stress on wound tissues, promoting angiogenesis and extracellular matrix remodeling, effects which are especially beneficial for such wounds.^33^ In contrast, purse-string closures involve smaller, lower-tension wounds, potentially limiting NPWT's additional benefits. Furthermore, NPWT's continuous subatmospheric pressure facilitates fluid drainage and microbial decontamination, which are more critical in linear closure wounds (where fluid accumulation and infection risks are inherently higher due to larger size). These mechanistic differences may explain why NPWT reduced wound complications in the linear closure group but not in the PSC group. However, due to limited sample size, these efficacy differences require validation via large-scale RCTs.

Notably, no significant differences in seroma and hematoma rates were observed, aligning with some studies^34^ but contrasting with others.^35^^,^^36^ Inconsistencies may stem from limited statistical power (small sample size) and methodological variability in intervention protocols (e.g., pressure settings, dressing replacement frequency). For nursing practice, this suggests NPWT may not reduce all types of wound complications, and clinical monitoring for seroma/hematoma should remain standard part of post-stoma reversal care.

### Implications for nursing practice and research

These findings highlight NPWT as a valuable intervention for reducing wound complications and optimizing recovery after stoma reversal, particularly in patients undergoing linear closure. Given the substantial global health burden and economic impact of wound complications, NPWT offers dual benefits: improving clinical outcomes and enhancing healthcare resource utilization.^37^ Accelerating wound healing and reducing hospital stays positions NPWT as a potential strategy for lowering healthcare costs, especially in resource-limited settings.^38^

Future research should prioritize large-scale, multicenter RCTs with extended follow-up to validate NPWT's differential efficacy across closure techniques and establish standardized protocols. Incorporating patient-centered outcomes (e.g., pain intensity, satisfaction, quality of life) and long-term endpoints (e.g., hernia rates) is crucial for comprehensive evaluation.

Regarding economic outcomes, while two included trials found no significant differences in direct medical expenditures between NPWT and control groups,^21^^,^^25^ this does not negate potential economic benefits. Our study confirmed a mean reduction in hospital stay of 1.05 days with NPWT, alleviating bed occupancy pressure and lowering indirect costs (e.g., nursing hours, auxiliary service). In resource-constrained environments, even modest reductions in hospitalization duration can improve resource allocation, enabling facilities to serve more patients and reducing the economic burden of prolonged care. Supporting this, prior evidence in colorectal surgery indicates NPWT may lower long-term costs by reducing complication rates in high-risk populations.^39^ Though our data showed no direct cost savings, the combined effect of shortened recovery time and potential complication reduction suggests NPWT holds economic value, particularly in cost-sensitive care pathways.

### Limitations

This study had several limitations. First, Only 8 studies were included, potentially reducing result reliability. Subgroup analyses (e.g., linear closure group, *n* ​= ​201) had limited power, possibly leading to false-positive conclusions.

Second, heterogeneity in NPWT protocols (pressure settings: −80 ​mmHg to −125 ​mmHg; treatment duration: 3–14 days) may have introduced outcomes variability. While sensitivity analyses suggested overall findings were robust, the lack of standardized protocols limits conclusions about optimal NPWT parameters for stoma reversal. Importantly, the meta-analysis evaluated data from a single NPWT device, limiting extrapolatio to other systems with different specifications (e.g., pressure range, exudate management mechanisms, dressing materials).

Moreover, the absence of patient-centered outcomes (e.g., pain, aesthetic outcomes, healthcare utilization) represents a significant gap, as these are critical for understanding NPWT's full impact on patient experience and quality of life.

Future studies should prioritize large-scale, multicenter RCTs with extended follow-up to address these gaps. They should standardize NPWT protocols (pressure, duration, device types), include diverse patients (e.g., those with complex wounds), measure patient-reported outcomes (pain, satisfaction) and long-term effects (e.g., hernia rates), and compare cost-effectiveness of NPWT systems.

## Conclusions

Our study demonstrates that NPWT in stoma reversal following colorectal surgery significantly reduces wound complications, accelerates wound healing, and shortens hospital stays compared to conventional dressings. Notably, these benefits were most pronounced in patients undergoing linear closure. Despite these advantages, the lack of standardized NPWT protocols and reliance on surgeon preferences limit its widespread adoption. These findings highlight NPWT's potential to improve clinical outcomes and reduce healthcare costs, particularly in resource-limited settings, where its efficiency in shortening hospital stays and minimizing complications could offer significant economic and patient benefits. Future high-quality, multicenter RCTs with standardized protocols are essential to establish NPWT as a routine intervention for stoma closure in colorectal surgery.

## CRediT authorship contribution statement

**Siqing Li:** conceptualization, methodology, resources, software, writing – original draft. **Xirong Huang and Xiaofang Guan:** Resources, investigation, data curation, and writing – original draft. **Zhong Wang:** methodology, software, formal analysis, and visualization. **Minyi Xie:** Investigation, funding acquisition, writing, review, and editing. **Linjie Mo:** methodology, investigation, validation, and resources. **Yuxia Liu** and **Wenxin Luo:** writing, review and editing, supervision, project administration, and funding acquisition. All authors have read and approved the final manuscript.

## Ethics statement

Not required.

## Data availability statement

Data are available on request from the authors. The data supporting the findings of this study are available from the corresponding authors (Wenxin Luo or Yuxia Liu) upon reasonable request.

## Declaration of generative AI and AI-assisted technologies in the writing process

No AI tools/services were used during the preparation of this work.

## Funding

This study was supported by the Social Development Program of the Zhuhai Science and Technology Bureau (Grant No. 2320004000239 and 2320004000209). The funders had no role in considering the study design or in the collection, analysis, interpretation of data, writing of the report, or decision to submit the article for publication.

## Declaration of competing interest

The authors declare no conflict of interest.
