# Supplementary material for: Negative pressure wound therapy for preventing wound complications following stoma reversal: A systematic review and meta-analysis of randomized controlled trials
Source: Asia Pac J Oncol Nurs. 2025 Aug 26;12:100778. doi: 10.1016/j.apjon.2025.100778 (PMC12590011; doi:10.1016/j.apjon.2025.100778)
Supplement: Multimedia component 1 [file mmc1.docx]

**Supplementary Table S1.** PubMed Search Trail (Search updated 01/12/2024)

| Search # | MeSH Terms and Key Words |
| --- | --- |
| #1 | "negative pressure wound therapy"[MeSH Terms] OR "negative pressure wound therapy"[Title/Abstract] OR "Negative-Pressure Wound Therapies"[Title/Abstract] OR "therapies negative pressure wound"[Title/Abstract] OR "therapy negative pressure wound"[Title/Abstract] OR "wound therapy negative pressure"[Title/Abstract] OR "topical negative pressure therapy"[Title/Abstract] OR "negative pressure therapy topical"[Title/Abstract] OR "therapy topical negative pressure"[Title/Abstract] OR "topical negative pressure therapy"[Title/Abstract] OR "negative pressure dressings"[Title/Abstract] OR "dressing negative pressure"[Title/Abstract] OR "dressings negative pressure"[Title/Abstract] OR "negative pressure dressings"[Title/Abstract] OR "Negative-Pressure Dressing"[Title/Abstract] OR "vacuum assisted closure"[Title/Abstract] OR "closure vacuum assisted"[Title/Abstract] OR "vacuum assisted closure"[Title/Abstract] OR "Vacuum-Assisted Closures"[Title/Abstract] |
| #2 | "ileostomy closure"[Title/Abstract] OR "ileostomy reversal"[Title/Abstract] OR "Ostomy Closure"[Title/Abstract] OR "stoma reversal"[Title/Abstract] OR "stoma closure"[Title/Abstract] OR "closure of ileostomy"[Title/Abstract] |
| #3 | "Randomized Controlled Trial"[Publication Type] OR "Randomized Controlled Trial"[Title/Abstract] OR "clinical trials randomized"[Title/Abstract] OR "trials randomized clinical"[Title/Abstract] OR "controlled clinical trials randomized"[Title/Abstract] OR "Controlled Clinical Trial"[Title/Abstract] OR "clinical trial"[Title/Abstract] OR "Randomly"[Title/Abstract] OR "randomized"[Title/Abstract] OR "RCT"[Title/Abstract] OR "RCTs"[Title/Abstract] |
| #4 | #1 AND #2 AND #3 |

**Supplementary Table S2.** Web of Science Search Trail (Search updated 01/12/2024)

| Search # | TS |
| --- | --- |
| #1 | ((((((((((((((((((((((TS=("Negative-Pressure Wound Therapy")) OR TS=("Negative Pressure Wound Therapy")) OR TS=("Negative-Pressure Wound Therapies")) OR TS=("Therapies, Negative-Pressure Wound")) OR TS=("Therapy, Negative-Pressure Wound")) OR TS=("Wound Therapies, Negative-Pressure")) OR TS=("Topical Negative-Pressure Therapy")) OR TS=("Negative-Pressure Therapies, Topical")) OR TS=("Negative-Pressure Therapy, Topical")) OR TS=("Therapies, Topical Negative-Pressure")) OR TS=("Therapy, Topical Negative-Pressure")) OR TS=("Topical Negative Pressure Therapy")) OR TS=("Topical Negative-Pressure Therapies")) OR TS=("Negative-Pressure Dressings")) OR TS=("Dressing, Negative-Pressure")) OR TS=("Dressings, Negative-Pressure")) OR TS=("Negative Pressure Dressings")) OR TS=("Negative-Pressure Dressing")) OR TS=("Vacuum-Assisted Closure")) OR TS=("Closure, Vacuum-Assisted")) OR TS=("Closures, Vacuum-Assisted")) OR TS=("Vacuum Assisted Closure")) OR TS=("Vacuum-Assisted Closures") |
| #2 | (((((TS=("ileostomy closure")) OR TS=("ileostomy reversal")) OR TS=("Ostomy Closure")) OR TS=("stoma reversal")) OR TS=("stoma closure")) OR TS=("closure of ileostomy") |
| #3 | (((((((((TS=("Randomized Controlled Trials")) OR TS=("Clinical Trials, Randomized")) OR TS=("Trials, Randomized Clinical")) OR TS=("Controlled Clinical Trials, Randomized")) OR TS=(RCT)) OR TS=("Controlled Clinical Trial")) OR TS=("clinical trial")) OR TS=(RCTs)) OR TS=(Randomly)) OR TS=(randomized) |
| #4 | #1 AND #2 AND #3 |

**Supplementary** **Table S3.** Embase Trail (Search updated 01/12/2024)

| Search # | MeSH Terms and Key Words |
| --- | --- |
| #1 | 'negative-pressure wound therapy':ab,ti OR 'negative pressure wound therapy':ab,ti OR 'negative-pressure wound therapies':ab,ti OR 'therapies, negative-pressure wound':ab,ti OR 'therapy, negative-pressure wound':ab,ti OR 'wound therapies, negative-pressure':ab,ti OR 'wound therapy, negative-pressure':ab,ti OR 'topical negative-pressure therapy':ab,ti OR 'negative-pressure therapies, topical':ab,ti OR 'negative-pressure therapy, topical':ab,ti OR 'therapies, topical negative-pressure':ab,ti OR 'therapy, topical negative-pressure':ab,ti OR 'topical negative pressure therapy':ab,ti OR 'topical negative-pressure therapies':ab,ti OR 'negative-pressure dressings':ab,ti OR 'dressing, negative-pressure':ab,ti OR 'dressings, negative-pressure':ab,ti OR 'negative pressure dressings':ab,ti OR 'negative-pressure dressing':ab,ti OR 'vacuum-assisted closure':ab,ti OR 'closure, vacuum-assisted':ab,ti OR 'closures, vacuum-assisted':ab,ti OR 'vacuum assisted closure':ab,ti OR 'vacuum-assisted closures':ab,ti |
| #2 | 'vacuum assisted closure'/exp |
| #3 | #1 OR #2 |
| #4 | 'ileostomy closure':ab,ti OR 'ileostomy reversal':ab,ti OR 'ostomy closure':ab,ti OR 'stoma reversal':ab,ti OR 'stoma closure':ab,ti OR 'closure of ileostomy':ab,ti |
| #5 | 'randomized controlled trial':ab,ti OR 'clinical trials, randomized':ab,ti OR 'trials, randomized clinical':ab,ti OR 'controlled clinical trials, randomized':ab,ti OR 'rct':ab,ti OR 'rcts':ab,ti OR 'controlled clinical trial':ab,ti OR 'clinical trial':ab,ti OR 'randomly':ab,ti OR 'randomized':ab,ti OR 'randomised':ab,ti OR 'randomization':ab,ti OR 'randomisation':ab,ti |
| #6 | #3 AND #4 AND #5 |

**Supplementary Table S4.** Cochrane Library Trail (Search updated 01/12/2024)

| Search # | MeSH Terms and Key Words |
| --- | --- |
| #1 | ileostomy closure OR ileostomy reversal OR Ostomy Closure OR stoma reversal OR stoma closure OR closure of ileostomy |
| #2 | Negative-Pressure Wound Therapy OR Negative Pressure Wound Therapy OR Negative-Pressure Wound Therapies OR Therapies, Negative-Pressure Wound OR Therapy, Negative-Pressure Wound OR Wound Therapies, Negative-Pressure OR Wound Therapy, Negative-Pressure OR Topical Negative-Pressure Therapy OR Negative-Pressure Therapies, Topical OR Negative-Pressure Therapy, Topical OR Therapies, Topical Negative-Pressure OR Therapy, Topical Negative-Pressure OR Topical Negative Pressure Therapy OR Topical Negative-Pressure Therapies OR Negative-Pressure Dressings OR Dressing, Negative-Pressure OR Dressings, Negative-Pressure OR Negative Pressure Dressings OR Negative-Pressure Dressing OR Vacuum-Assisted Closure OR Closure, Vacuum-Assisted OR Closures, Vacuum-Assisted OR Vacuum Assisted Closure OR Vacuum-Assisted Closures |
| #3 | MeSH descriptor: [Negative-Pressure Wound Therapy] explode all trees |
| #4 | #2 OR #3 |
| #5 | MeSH descriptor: [Randomized Controlled Trial] explode all trees |
| #6 | Randomized Controlled Trial OR Clinical Trials, Randomized OR Trials, Randomized Clinical OR Controlled Clinical Trials, Randomized OR RCT OR RCTs OR Controlled Clinical Trial OR clinical trial OR Randomly OR Randomized OR Randomised OR Randomization OR Randomisation |
| #7 | #5 OR #6 |
| #8 | #1 AND #4 AND #7 |

**Supplementary Table S5.** Scopus Search Trail (Search updated 01/12/2024)

| Search # | Key Words |
| --- | --- |
| #1 | (TITLE-ABS-KEY("Negative Pressure Wound Therapy") OR TITLE-ABS-KEY("Therapies, Negative-Pressure Wound") OR TITLE-ABS-KEY("Wound Therapies, Negative-Pressure") OR TITLE-ABS-KEY("Topical Negative-Pressure Therapy") OR TITLE-ABS-KEY("Negative-Pressure Therapies, Topical") OR TITLE-ABS-KEY("Therapies, Topical Negative-Pressure") OR TITLE-ABS-KEY("Negative-Pressure Dressings") OR TITLE-ABS-KEY("Dressing, Negative-Pressure") OR TITLE-ABS-KEY("Vacuum-Assisted Closure") OR TITLE-ABS-KEY("Closure, Vacuum-Assisted")) |
| #2 | (TITLE-ABS-KEY("ileostomy closure") OR TITLE-ABS-KEY("ileostomy reversal") OR TITLE-ABS-KEY("Ostomy Closure") OR TITLE-ABS-KEY("stoma reversal") OR TITLE-ABS-KEY("stoma closure") OR TITLE-ABS-KEY("closure of ileostomy")) |
| S3 | (TITLE-ABS-KEY("Randomized Controlled Trial") OR TITLE-ABS-KEY("Clinical Trials, Randomized") OR TITLE-ABS-KEY("Trials, Randomized Clinical") OR TITLE-ABS-KEY("Controlled Clinical Trials, Randomized") OR TITLE-ABS-KEY("Controlled Clinical Trial") OR TITLE-ABS-KEY("clinical trial") OR TITLE-ABS-KEY(RCT) OR TITLE-ABS-KEY(RCTs) OR TITLE-ABS-KEY(Randomly) OR TITLE-ABS-KEY(Randomized)) |
| S4 | #1 AND #2 AND #3 |

**Supplementary Table S6.** China National Knowledge Infrastructure (CNKI) Search Trail (Search updated 01/12/2024)

| Search # | Key Words |
| --- | --- |
| #1 | （主题：负压伤口治疗 + 负压伤口治疗技术 + '负压伤口治疗(npwt)' + 负压伤口治疗仪 + 负压伤口治疗装置）OR（主题：负压封闭引流 + 负压封闭引流技术 + 负压封闭引流术 + '负压封闭引流(vsd)' + 持续负压封闭引流治疗 + 持续负压封闭引流技术）OR（主题：负压辅助闭合 + 负压辅助闭合技术 + 负压辅助闭合装置 + 负压辅助闭合创口技术） |
| #2 | （主题：造口回纳）OR（主题：造口还纳 + 造口还纳术 + 造口还纳手术）OR（主题：肠造口关闭）OR（主题：肠造口闭合 + 肠造口闭合术 + 肠造口闭合术后） |
| S3 | #1AND #2 |

**Supplementary Table S7.** Wanfang Search Trail (Search updated 01/12/2024)

| Search # | Key Words |
| --- | --- |
| #1 | 主题:(负压伤口治疗) or 主题:(负压封闭引流) or 主题:(负压辅助闭合) |
| #2 | [主题:(造口还纳) or 主题:(造口回纳) or 主题:(肠造口关闭) or 主题:(肠造口闭合)](https://s.wanfangdata.com.cn/advanced-search/paper?q=%E4%B8%BB%E9%A2%98:(%E9%80%A0%E5%8F%A3%E8%BF%98%E7%BA%B3) or %E4%B8%BB%E9%A2%98:(%E9%80%A0%E5%8F%A3%E5%9B%9E%E7%BA%B3) or %E4%B8%BB%E9%A2%98:(%E8%82%A0%E9%80%A0%E5%8F%A3%E5%85%B3%E9%97%AD) or %E4%B8%BB%E9%A2%98:(%E8%82%A0%E9%80%A0%E5%8F%A3%E9%97%AD%E5%90%88)&searchtype=expert&type=["periodical","thesis","conference"]&topicExpand=true" \t "https://s.wanfangdata.com.cn/advanced-search/_blank) |
| S3 | #1 AND #2 |

**Supplementary Table S8.** Chinese Biomedical Literature Database (CBM) Search Trail (Search updated 01/12/2024)

| Search # | MeSH Terms and Key Words |
| --- | --- |
| #1 | "肠造口关闭)"[全部字段:智能] OR "肠造口闭合"[全部字段:智能] OR "造口回纳"[全部字段:智能] OR "造口还纳"[全部字段:智能] |
| #2 | "局部负压疗法"[全部字段:智能] OR "负压包扎"[全部字段:智能] OR "负压封闭技术"[全部字段:智能] OR "负压封闭引流"[全部字段:智能] OR "负压辅助闭合"[全部字段:智能] OR "负压伤口治疗"[全部字段:智能] |
| #3 | "负压伤口疗法"[不加权:扩展] |
| #4 | #2 OR #3 |
| #5 | #1 AND #4 |
